# Effect of GA-sensitivity on wheat early vigor and yield components under deep sowing

**DOI:** 10.3389/fpls.2015.00487

**Published:** 2015-07-10

**Authors:** Avishay Amram, Aviya Fadida-Myers, Guy Golan, Kamal Nashef, Roi Ben-David, Zvi Peleg

**Affiliations:** ^1^The Robert H. Smith Institute of Plant Sciences and Genetics in Agriculture, The Hebrew University of JerusalemRehovot, Israel; ^2^Institute of Plant Sciences, Agricultural Research Organization – Volcani CenterBet Dagan, Israel

**Keywords:** coleoptile length, erratic precipitation pattern, gibberellin, semi-dwarf wheat, seedling establishment, *Rht*, yield components

## Abstract

Establishment of seedlings is a key factor in achievement of uniform field stands and, consequently, stable yields. Under Mediterranean conditions, soil moisture in the upper layer is limited and seedlings may be exposed to frequent dehydration events. The presence of the *Reduced height* (*Rht)-B1b* and *Rht-D1b* semi-dominant dwarfing alleles results in insensitivity to gibberellin (GAI) and, hence, poor emergence from deep sowing. Introduction of alternative dwarfing genes and, thereby, preservation of the gibberellin response (GAR) and coleoptile length, contributes to better emergence from deep sowing. Initially 47 wheat cultivars carrying different *Rht* alleles were screened for their ability to emerge from deep sowing, and then 17 of them were selected for detailed physiological characterization in the field. The modern wheat lines containing GAI alleles showed significantly lower percentages of emergence from deep sowing than the GAR lines, i.e., 52 and 74%, respectively. Differences in early developmental stages were associated with grain yield, as indicated by a reduction of 37.3% in the modern GAI cultivars. Our results demonstrate the potential of alternative dwarfing genes for improving seedling establishment and grain yields in Mediterranean-like environments.

## Introduction

Initial germination of seeds, and ability of seedlings to emerge and establish are key factors in achievement of uniform, high-yielding field stands. Soil moisture is necessary for seed germination and seedling growth but, under semi-arid Mediterranean conditions soil moisture in the upper layer is limited and seeds may be exposed to frequent dehydration events. Moreover, in recent years, climate change has led to increased fluctuation of precipitation (Marvel and Bonfils, 2013). However, deep sowing could ensure adequate seed-zone moisture before germination and thereby enhance seedling establishment (Richards et al., 2010; Mohan et al., 2013; Rebetzke et al., 2014b). Deep sowing could also be important for avoiding the phytotoxicity of pre-emergence herbicides (O’Sullivan et al., 1985) and preventing seed removal by predators (Brown et al., 2003).

Since the early 1970s the ‘Green Revolution’ has led to a significant improvement in wheat yields, through incorporation of *Reduced height* (*Rht*) alleles (Gale and Youssefien, 1985) to produce semi-dwarf cultivars (Flintham et al., 1997). *Rht-B1* and *Rht-D1* (formerly *Rht1* and *Rht2*) encode DELLA proteins (named after their conserved N-terminal D-E-L-L-A amino acid motif), which act as repressors of gibberellin (GA)-responsive growth. The *Rht-B1b* and *Rht-D1b* mutations are gain-of-function mutations that impair GA signaling and thereby confer dwarfism through constitutive repression of cell division and elongation (Peng et al., 1999). The shortened stem of GAI wheat results in liberation of more assimilates to the parallel process of spike development, thereby contributing to harvest index (HI) improvement and, consequently, increased total grain yield (Austin et al., 1980). The *Rht* improves lodging resistance and enables increased application rates of chemical fertilizers.

However, modern GAI wheat cultivars have short coleoptiles (the sheath-like structure that protects the elongating seedling as it emerges through the soil surface), therefore they will not establish well if sown too deep (Allan, 1980; Supplementary Figure S1). In attempting to counter these problems of establishment under deep sowing, several alternative dwarfing genes that confer *Rht* while retaining responsiveness to endogenous gibberellin (GAR) have been characterized (Ellis et al., 2004; Rebetzke et al., 2011, 2014b; Chen et al., 2013).

The use of alternative dwarfing genes contributed to the development of elite wheat cultivars with longer coleoptiles than those of GAI cultivars, and improved establishment from deep sowing (Schillinger et al., 1998). Lines containing these genes emerge more successfully than those without them, when sown deep or when used in conservation tillage systems (Rebetzke et al., 2012). Enhanced responsiveness to GA could also contribute to improved early vigor and could reduce water loss from the soil and thereby improve competitiveness with weeds. In the present study, we evaluated the breeding potential of alternative GAR dwarfing genes under semi-arid Mediterranean conditions. The aims were: (*i*) morpho-physiological characterization of GA responsiveness in a collection of Mediterranean wheat cultivars; (*ii*) measurement of the effect of GA responsiveness on emergence ability from deep sowing; and (*iii*) evaluation of the effect of GA responsiveness on yield components under deep sowing.

## Materials and Methods

### Plant Material

Forty-seven wheat genotypes carrying different dwarfing alleles were screened for their ability to emerge from deep sowing. The germplasm array included: ten modern Israeli cultivars carrying green-revolution-derived dwarfing alleles (*Rht-B1b* and *Rht-D1b*); 34 Mediterranean wheat landraces, and three wheat cultivars carrying known GAR genes (*Rht8, Rht5, Rht13*; Supplementary Table S1). Following the initial screening, a subset of 17 wheat cultivars (seven modern cultivars, eight landraces, and two wheat lines carrying known GAR genes) were selected for detailed physiological characterization. For each genotype the year of collection or cultivar release, and molecular characterization data are given in **Table 1**.

**Table 1:** Wheat cultivars used for the field experiment, with years of release or collection and descriptions of *Rht* genes.

Genotype/cultivar	ID	Year	Country	*Rht genes*
**GA-responsive genotypes**				
Abu Fashi	PI 384037	1937	Israel	*Rht12*
Chuan Mai-18	–	1965	China	*Rht8*
Fere Alexandrinum	PI 134596	1939	Syria	*Rht9, Rht12*
Gaza	Cltr 12616	1947	Israel	*Rht9*
Juljulith	PI 292034	1936	Israel	–
JM 3989	PI 572903	1991	Palestine	*Rht12, Rht13*
Langdon	Cltr 13165	1954	USA	*Rht5*
Marfed Dwarf	PI 542439	1987	USA	*Rht5, Rht13*
MG 26427	PI 534500	1988	Egypt	–
Persian Black	PI 283891	1926	Iran	*Rht5*
**GA-insensitive genotypes**				
C-61		2013	Israel	*Rht-B1b, Rht5, Rht13*
Bet Hashita		1985	Israel	*Rht-B1b, Rht12, Rht13*
Zahir		2005	Israel	*Rht-B1b, Rht8, Rht12, Rht13*
Yuval		2012	Israel	*Rht-B1b, Rht12*
Nirit		1996	Israel	*Rht-B1b, Rht9*
Omer		2013	Israel	*Rht-B1b, Rht5, Rht8, Rht12*
Shoam		2003	Israel	*Rht-D1b, Rht12*

### Characterization of Emergence from Deep Sowing

**FIGURE 1:**
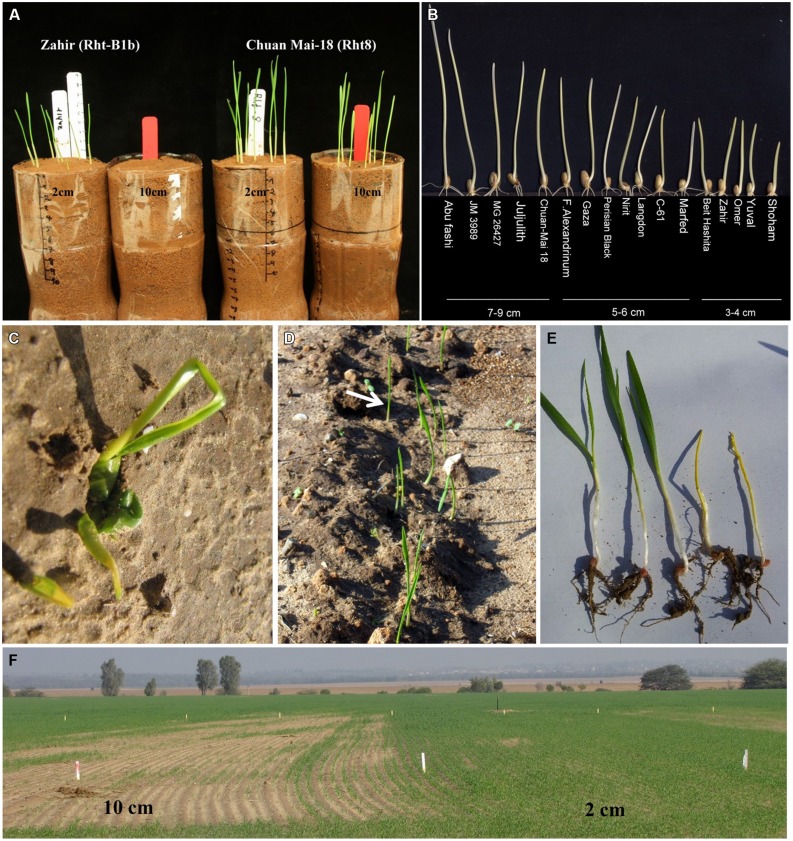
**(A)** Example of the laboratory screening for emergence from deep sowing. **(B)** Coleoptile length distribution among 17 wheat genotypes; photo was taken 7 days after sowing. **(C)** Example of leaf emergence from deep sowing. **(D)** Coleoptile (marked with arrow) emergence from deep sowing. **(E)** Effects of deep sowing on modern gibberellin-insensitive cultivars. **(F)** Field experiment of deep sowing compared with common sowing practice.

Emergence from deep sowing was conducted in (9 × 20)-cm cylindrical plastic containers filled with brown-red degrading sandy soil (Rhodoxeralf) composed of 76% sand, 8% silt and 16% clay (**Figure 1A**). Eight uniform-size seeds were placed, at 2 or 10 cm depth, in the control or deep-sowing treatments, respectively, and watered to field capacity. The pots were placed in a dark room at 18°C and were monitored daily for number of emerging seedlings. The experiment was repeated three times.

In addition, we tested the effect of soil type (Rhodoxeralf soil and heavy clay soil) and sowing depth (2 or 10 cm) on plant emergence of GAI (Israeli cultivars Yuval and Zahir) and GAR (wheat lines Marfed Dwarf and Chuan Mai-18) wheat genotypes.

### Coleoptile Length Measurement

The screening system was based on the ‘cigar roll’ method (Bai et al., 2013). Ten uniform-size seeds of each genotype were placed on moist germination paper (25 cm × 38 cm; Anchor Paper Co., St. Paul, MN, USA), about 4 cm apart, with germ end down. The paper was covered with another sheet of moist germination paper and the sandwich was rolled to a final diameter of 2 cm. The bases of the rolls were placed in water in a tray in a darkened growth chamber at a constant temperature of 18°C. After 7 days, the average coleoptile length of eight seedlings was recorded, to the nearest millimeter, as measured from the base of the seed to the coleoptile tip.

### GA-Sensitivity Assay

Uniform-size seeds of each genotype were rolled in moist germination paper, according to the ‘cigar roll’ method (Bai et al., 2013). The base of the rolls were placed in a tray with water (control) or 10^-5^ M gibberellic acid (GA_3_; Cat. No. G-7645; Sigma; GA-treatment) and placed in a darkened growth chamber at a constant temperature of 10°C. Coleoptile lengths were measured 10 days after germination.

### Molecular Analysis

Fresh leaf tissue (∼50 mg) from individual 6 days-old seedling were used for DNA extraction by the CTAB method, follow RNase treatment. A NanoDrop ND1000 spectrophotometer (NanoDrop Technologies, Wilmington, DE, USA) was used to measure the DNA concentration. Polymerase chain reaction (PCR) protocols and primers used in this study were as described by Ellis et al. (2002, 2005). Fragment sizes of PCR products were determined on 2% agarose gel and visualized under UV light after staining with ethidium bromide. The primer pairs for each *Rht* gene were: *Rht-B1b* – BF and WR1; *Rht-D1b* – BF2 and WR2 (Ellis et al., 2002); *Rht5* – *XBarc102*; *Rht8 – Xwmc503*; *Rht9 – XBarc151*; *Rht12 – Xwmc410*; *Rht13 – Xwms577* (Ellis et al., 2005).

### Field Trial for Emergence from Deep Sowing

A field experiment was conducted at the experimental farm of the Hebrew University of Jerusalem in Rehovot, Israel (34°47′ N, 31°54′ E; 54 m above sea level). A split-plot factorial (genotype/sowing depth) block design with six replicates was employed; each block consisted of two main plots (one for each of the two sowing depths), with each main plot split into 17 single-row plots (one for each genotype). The plants were spaced at 20 and 30 cm, within and between rows, respectively. Two sowing-depth treatments were used: 2-cm as control, to mimic the common agro-technical practice; and 10-cm deep-sowing treatment. The field was treated with fungicides and pesticides to prevent development of fungal pathogens or insect pests, and was weeded manually weekly.

The number of seedlings that emerged from each soil depth was recorded. Fifty-one days after sowing, the number of leaves on the main stem was recorded. Sixty days after sowing, the number of tillers and width of the last fully exposed leaf were determined. The heading date of each plot, i.e., when the first spikes of 50% of the plants were fully exposed, was recorded. The thousand-kernel weight (TKW) for each plot was determined by using an electronic seed counter (Model S-25; DATA Technologies, Ltd., Jerusalem, Israel) and a digital scale. Numbers of spikes per plant, spikelets per spike, and grains per spikelet, and total grain yield were measured and calculated on a single-plot basis.

### Statistical Analyses

The JMP statistical package, Version 11 (SAS Institute Inc., Cary, NC, USA) was used for statistical analyses. Principal component analysis (PCA) of the eight continuous plant traits was used to identify a smaller number of principal components that accounted for most of the phenotypic variance found in the field assay. The PCA was based on the correlation matrix and is presented as biplot ordinations of wheat lines (PC scores) and continuous plant traits (PC factor loading). Two components were extracted by using eigenvalues >1 to ensure meaningful implementation of the data by each factor.

## Results

### Physiological Characterization of the Wheat Germplasm

We observed wide phenotypic diversity among the 47 tested wheat genotypes, in their ability to emerge from deep-sowing (10 cm; Supplementary Table S1). Based on this initial screening (**Figure 1A**), we selected for detailed characterization 17 genotypes that represented the variation in emergence from depth. It is worth noting that whereas in the early 19th century most wheat cultivars, i.e., landraces, grown in this region were of durum wheat, since the mid-20th century those cultivars were replaced with bread wheat and, subsequently, with the GAI “green revolution” cultivars. Therefore, our selection of genotypes for the present study represents the material grown by farmers in this region during the last century. Among these lines, wide variations in coleoptile length were detected (**Figure 1B**). It is important to note that when GAI wheat genotypes with short coleoptiles are used in deep sowing the first leaf is forced to penetrate the soil upper layers. As observed later in the current field experiment, this could result in slow emergence and might cause physical damage to the developing young tissue (**Figures 1C–F**).

**FIGURE 2:**
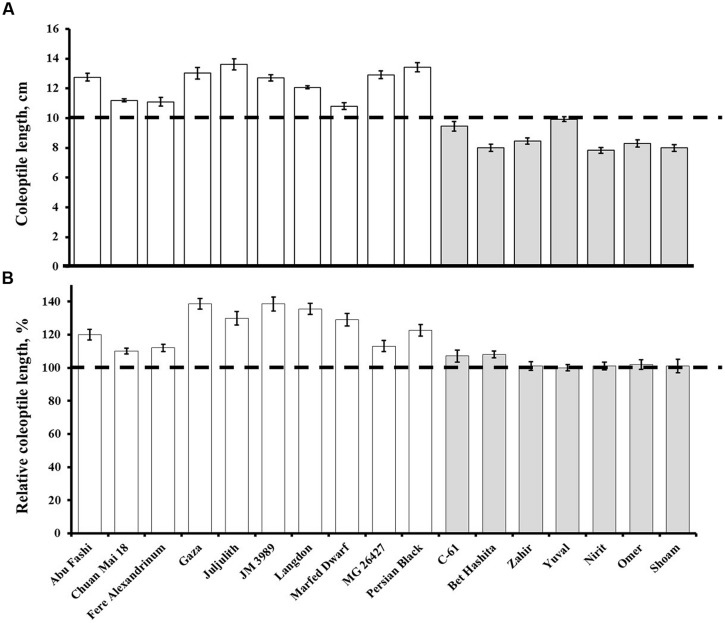
**(A)** Coleoptile lengths of the gibberellin-responsive (GAR; white) and GA-insensitive (GAI, gray) genotypes under controlled conditions. Dashed line marks the sowing depth. Data are means ± SE (*n* = 10). **(B)** Effect of exogenous application of GA on coleoptile length. Data presented as percentages of control values. Dashed line indicates no change, i.e., 100%. Data are means ± SE (*n* = 10).

Under controlled conditions, the coleoptile length of the wheat germplasm ranged from 7.84 cm (Nirit) to 13.62 cm (Juljulith), with an average of 10.80 cm (**Figure 2A**; **Table 2**). The coleoptile length of GAI cultivars was significantly (*P* = 0.0001) shorter than that of the GAR cultivars, at 8.57 and 12.36 cm, respectively. It is worth noting that all the GAI cultivars had coleoptiles that were shorter than the present sowing depth of 10 cm, hence only leaf was emerging through the soil surface. The average time to emergence from deep-sowing was significantly (*P* = 0.0001) longer for the GAI than for the GAR lines: 11.87 and 9.16 days, respectively. The GAI lines showed non-significant responses to application of exogenous GA (**Figure 2B**; gray column). In contrast, several GAR lines showed significant increments in coleoptile length in response to GA (**Figure 2B**; white column).

**Table 2:** Phenotypic and agronomic characterization of the 10 gibberellin-responsive (GAR) genotypes and seven modern gibberellin-insensitive (GAI) cultivars under controlled laboratory conditions and under field conditions. Field data represent the relative performance of the genotype under deep sowing treatment (10 cm) vs. control (2 cm).

	Pots experiment			Field experiment													
	Emergence	Days to emergence	Coleoptile length (cm)	Emergence	Number of tillers	Number of leaves	Leaf width	Effect on days to heading	Spikes per plant	Spikelets per spike	Grains per spike	Grains per spikelet	Grains per plant	Dry weight per plant	Harvest index	Thousand kernel weight	Grain yield
**GAR genotypes**				
Abu Fashi	95.8	9.8	12.8	64.7	72.6	99.6	90.3	1.9	88.3	104.8	90.6	104.4	80.4	81.4	101.0	102.6	53.7
Chuan Mai–18	109.1	10.1	11.2	80.3	34.9	94.9	105.7	-1.5	81.9	106.4	97.4	91.6	79.4	82.1	95.2	97.9	62.5
Fere Alexandrinum	83.3	9.9	11.1	70.6	40.6	83.8	91.0	1.9	74.1	95.3	94.0	98.1	70.5	68.5	107.0	104.2	51.8
Gaza	95.7	9.5	13.0	70.4	52.3	93.0	95.2	-0.4	80.5	96.5	116.9	120.9	90.5	85.9	111.4	103.1	64.9
Juljulith	104.8	8.5	13.6	64.3	51.7	92.3	100.0	0.5	91.3	98.5	91.4	93.6	80.7	85.5	94.0	100.4	51.8
JM 3989	104.4	8.9	12.7	90.0	65.3	92.1	92.7	1.7	77.0	89.8	93.8	104.3	72.3	68.3	105.7	98.8	64.4
Langdon	100.0	9.3	12.1	80.0	60.3	96.1	96.0	0.0	86.7	106.0	98.2	92.1	85.7	85.0	99.4	100.7	68.6
Marfed Dwarf	100.0	10.3	10.8	82.8	69.1	93.8	89.2	0.0	92.0	94.8	107.0	113.2	97.5	91.8	114.0	105.6	84.5
MG 26427	83.3	8.4	12.9	73.3	25.2	91.8	99.2	1.4	93.8	107.9	118.8	110.4	114.8	113.0	107.0	105.6	89.4
Persian Black	95.8	8.4	13.4	63.3	56.9	97.6	95.7	0.0	110.9	94.8	105.5	112.1	107.5	108.4	110.1	101.4	69.5
**GAI cultivars**				
C-61	95.0	11.7	9.5	49.1	18.9	86.6	81.9	1.7	57.2	91.1	88.0	96.4	49.8	54.5	94.8	102.5	25.2
Bet Hashita	104.6	11.4	8.0	41.7	32.4	75.2	60.1	5.0	66.3	91.1	90.5	101.4	72.6	70.7	89.7	97.1	29.4
Zahir	79.2	11.0	8.5	43.3	55.7	96.4	89.1	1.7	96.6	97.9	84.0	87.2	63.9	75.0	88.4	100.4	28.3
Yuval	87.0	10.9	9.9	49.1	51.7	83.1	82.5	3.0	53.6	99.5	79.0	80.0	58.2	51.4	99.3	98.4	27.8
Nirit	81.8	11.3	7.8	55.6	49.9	88.3	80.4	3.4	59.6	95.7	91.1	99.0	57.5	63.8	87.1	100.0	32.3
Omer	100.0	11.7	8.3	73.0	82.4	89.8	93.4	1.4	71.1	93.0	118.2	127.9	82.9	81.3	102.0	100.7	61.0
Shoam	109.5	11.8	8.0	52.2	7.4	78.1	74.9	2.8	62.1	82.4	86.3	104.3	53.9	53.6	100.8	102.9	28.9

Genotype^a^	46.4	18.2	59.1	1985.5	433.4	14.5	947.8	19.1	1819.0	175.0	442.4	87.7	2622.9	2113.9	404.6	12.9	2971.1
P(F)<	n.s.	^∗∗∗^	^∗∗∗^	^∗∗∗^	n.s.	n.s.	^∗∗^	^∗∗^	^∗∗^	^∗^	n.s.	n.s.	^∗∗^	^∗∗^	^∗∗^	n.s.	^∗∗^

*ANOVA results for each variable are presented in the bottom rows.*

*^a^Mean square of the genotypic group factor (GAI/GAR).*

*^∗^, ^∗∗^, ^∗∗∗^ and n.s. indicate significance level of P ≤ 0.05, 0.01, 0.001, and non-significant, respectively.*

### Genetic Characterization of the Wheat Germplasm

Characterization of the wheat germplasm with known DNA markers for *Rht* genes showed that all the modern cultivars possessed “marker alleles” for the dominant dwarfing gene: *Rht-B1b* in C-61, Bet Hashita, Zahir, Yuval, Nirit, and Omer; or *Rht-D1b* in Shoam. The DNA-marker analysis demonstrated that these lines also contained the dwarfing alleles in the loci linked to other dwarfing genes (GAR) (**Table 1**). The wheat landraces contained markers for several known dwarfing alleles in the alternative *Rht* loci: *Rht5* in Langdon, Marfed Dwarf, and Persian Black; *Rht8* in Chuan Mai-18; *Rht9* in Fere Alexandrinum, and Gaza; *Rht12* in Abu Fashi, Fere Alexandrinum, and JM 3989; and *Rht13* in JM 3989 and Marfed Dwarf. In Juljulith and MG 26427 no known markers associated with dwarfing genes were found (**Table 1**).

### Effect of Sowing Depth on Emergence and Field Establishment

**FIGURE 3:**
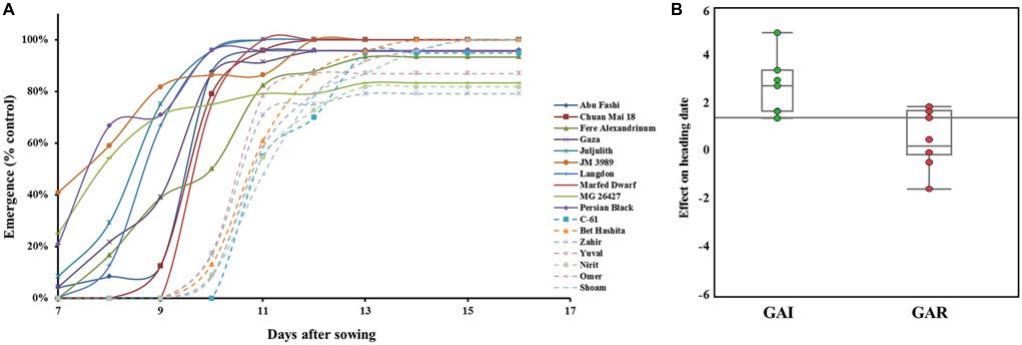
**Effects of sowing depth on seedling emergence and heading dates. (A)** Seedling emergence percentages from deep sowing as compared with control sown at 2 cm. Dashed lines represent GAI-genotypes; solid lines GAR-genotypes; **(B)** A box plot showing the effect of deep sowing on heading date as compared with control under field conditions. Components of descriptive statistics are graphically presented: median value (horizontal short line), quartile range (rectangle), data range (vertical long line).

When sown deep, the modern GAI wheat lines showed significantly (*P* = 0.0002) lower emergence percentages than the GAR lines, averaging 52 and 74%, respectively. However, it is worth noting that one GAI line – cv. Omer – showed a high emergence percentage of 73% (**Table 2**). These GAI lines also showed lower emergence percentages than the GAR lines under controlled conditions in the field (**Figure 3A**). Under deep sowing, the percentage reductions in leaf width, compared with the control, were significantly (*P* = 0.0007) higher in the GAI lines than in the GAR lines, averaging 19.7 and 4.5%, respectively (Supplementary Figure S2). Likewise, the reduction in the number of leaves was significantly (*P* = 0.0005) higher in the GAI than in the GAR lines, with averages of 14.7 and 6.5%, respectively (Supplementary Figure S2). The lower emergence rate from deep sowing was associated with delay flowering of the GAI lines – by 2.5 days (**Figure 3B**). This finding was also reflected in a strong correlation between emergence time and heading date (*r* = 0.55, *P* = 0.027).

Clear interactions were detected between genotypes and soil types. The Israeli cultivars that are characterized by short coleoptiles (**Figure 1B**) exhibited significantly slower emergence from depth in both soil types (Supplementary Figure S3A). In contrast, in the case of Marfed Dwarf, which contains the *Rht*5 GAR gene, significant differences in emergence rate were detected only in heavy clay soil but not in light sandy soil (Supplementary Figure S3B). However, soil type did not significantly affect the emergence performances of Chuan Mai-18, which contains the *Rht*8 GAR gene; this wheat line expressed high early vigor and emergence rates at both sowing depths and in both soil types.

### Effects of Sowing Depth on Grain Yield and Yield Components

**FIGURE 4:**
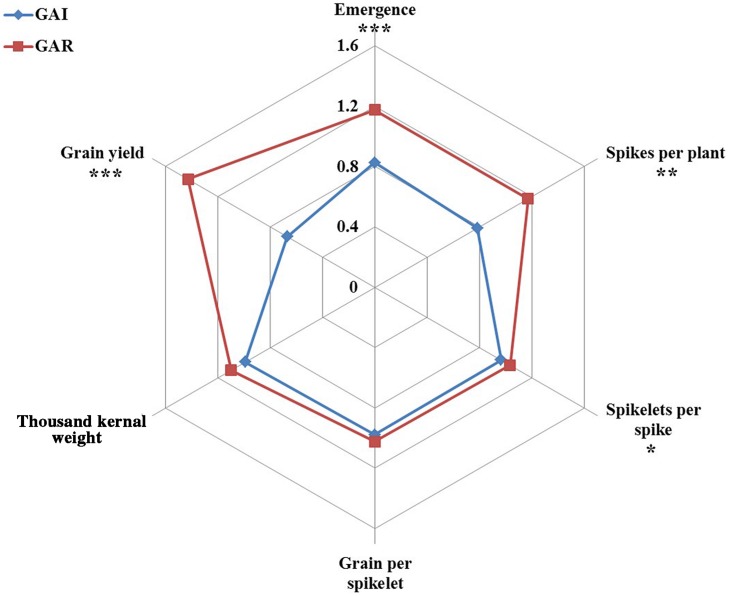
**Radar charts comparing the traits of yield and yield components of GAI (blue) and GAR (red) plants under deep sowing.** Six individual plots per line were used in each of the measurements, and the data were subjected to one-way ANOVA followed by Student’s *t*-test. Asterisks indicate significance: ^∗^*P* ≤ 0.05, ^∗∗^*P* ≤ 0.01, and ^∗∗∗^*P* ≤ 0.001.

The effects of deep sowing were stronger on the GAI lines than on the GAR lines, for all yield components; the percentage reduction in the number of spikes per plant, i.e., fertile tillers, was significantly (*P* = 0.002) lower in GAR than in GAI, at 12.3 and 33.4%, respectively (**Figure 4**; **Table 2**). Whereas the GAR lines were not affected by deep sowing, the GAI lines showed significantly (*P* = 0.02) fewer spikelet per spike, by 7.5% (**Figure 4**). The final grain yield was significantly affected by deep sowing for all the wheat lines: the GAI lines showed a significant (*P* = 0.0001) 66.7% reduction in GY, and the GAR lines a 33.9% reduction (**Figure 4**; **Table 2**).

**FIGURE 5:**
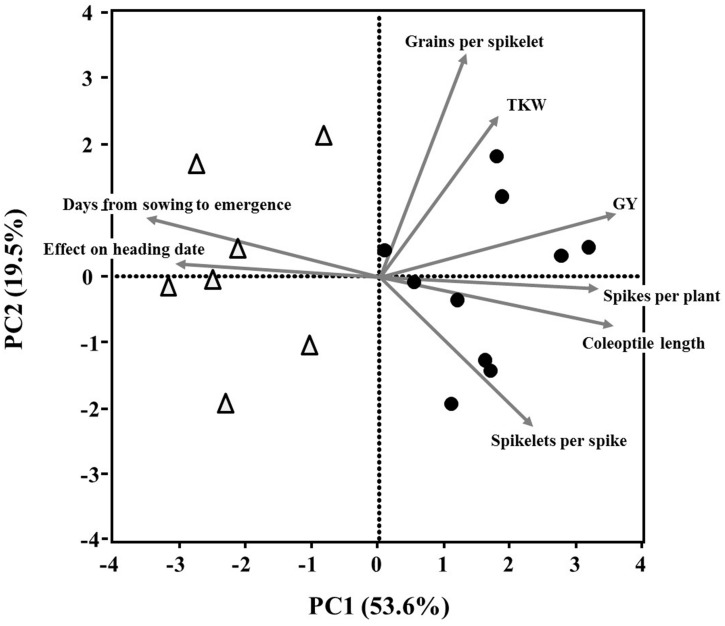
**Principal component analysis (based on correlation matrix) of continuous plant traits recorded on 17 wheat lines.** Biplot vectors are trait factor loadings for principal component (PC) 1 and PC2. The GAI lines (Δ) and GAR (●) are marked.

Principal component analysis extracted two major components (Eigenvalues >1) that accounted collectively for 73.1% of the variance in the data set. Principal component 1 (PC1, *X*-axis, **Figure 5**) explained 53.6% of the variation among lines, and was loaded positively with coleoptile length, spikes per plant and grain yield and negatively with days from sowing to emergence and effect on heading date. PC 2 (**Figure 5**; *Y*-axis) accounted for 19.5% of the variation; it was positively loaded with grains per spikelet and TKW, and negatively loaded with spikelets per spike. The PCA showed strong associations between days from sowing to emergence and heading date (**Figure 5**) – a finding supported by the positive correlations between these variables (*r* = 0.54, *P* = 0.002; Supplementary Table S2). The resulting PCA surface was able to distinguish between the GAI and the GAR lines, mainly evident along PC1 (**Figure 5**).

## Discussion

Under Mediterranean-like environments, the common sowing practice of the semi dwarf wheat lines containing the *Rht-B1b* and *Rht-D1b* exposes the seedlings to dehydration risk because of the high fluctuations in precipitation (Schillinger et al., 1998). To avoid this negative effects of these widely used dwarfing genes, several alternative genes (GAR) have been suggested as possible means to improve emergence from deep sowing (Ellis et al., 2004; Rebetzke et al., 2007, 2011, 2014b; Chen et al., 2013). In this context, it should be noted that the set of wheat landraces used in the present study had longer coleoptiles than the modern GAI cultivars, which contributed to their better emergence from deep sowing (**Figures 1–3**).

The ability of seedlings to emerge from soil depth and to establish a good field stand is considered the single most important phenological factor influencing the success of an annual plant (Forcella et al., 2000). This ability directly affects important parameters of early vegetative growth, such as number of leaves and leaf width, which determine subsequent photosynthesis and growth (Pang et al., 2014). Likewise, the number of spikelets per spike is determined during early development stages, and could be affected by emergence difficulties (Rebetzke et al., 2007). The use of short-coleoptile wheat genotypes in deep sowing or conservation cropping practices forces the developed young leaf to penetrate the soil upper layers. This negatively affects the physical properties of the seedling and its developmental processes: long-term research, which tested the performance of modern wheat cultivars in conservation cropping in Australia, estimated an 11% reduction in grain yield, which it attributed to poor establishment and poor early vigor as the main causes (Watt et al., 2006).

A longer coleoptile is an asset in promoting seedling emergence and establishment in drought-prone environments, where there is a lack of moisture in the top layer of the soil. Moreover, soil surface crusting, which is common under the Mediterranean fluctuating precipitation, necessitates fast coleoptile emergence (Schillinger et al., 1998), such as characterizes several landraces – Persian Black, Juljulith, JM 3989 and MG 26427 (**Figure 3A**).

The coleoptile has a dual role in both providing mechanical support, which is required for seedling emergence through the soil, and protecting the protruding leaf. Generally, it has been shown that GAI semidwarf genotypes have 30–40% shorter coleoptiles than traditional genotypes (Allan et al., 1962). In agreement with these findings, the modern cultivars in the present study showed significantly 30% shorter coleoptiles than the landrace genotypes (**Table 2**). Coleoptile length has previously been reported to be important for seedling emergence from deep sowing (Fick and Qualset, 1976; Richards et al., 2010), and in the present study it was significantly correlated with number of emerged seedlings (*r* = 0.59, *P* = 0.01; Supplementary Table S2) and, most importantly, with days to seedling emergence (*r* = -0.94, *P* = 0.0001; Supplementary Table S2).

Several environmental factors are considered to affect emergence, and they might interact with coleoptile length to determine early vigor of wheat seedlings. Among these factors are soil texture, seed-zone water content, and temperature (Jessop and Stewart, 1983; Mohan et al., 2013). In the present study we demonstrated the soil type × genotype interaction in a germination assay under controlled conditions (Supplementary Figure S3). Our results reflect the more challenging nature of the medium with which heavy clay soil confronts the emerging seedling, compared with that of the more favorable light sandy soil. Unlike Mohan et al. (2013), we do not dismiss the role of coleoptile length as a contributor to early vigor of wheat and its emergence from depth. Nevertheless, germination rate is the final outcome of the interaction of the wheat with soil-related environmental factors (Supplementary Figure S3).

Landraces have also shown higher values of leaf width and leaf number than the GAI cultivars and, therefore, greater early vigor when sown deep (Supplementary Figure S2). Leaf width was previously reported as a reliable indicator for early vigor, which is advantageous under Mediterranean or semiarid environments characterized by a short growing season (Rebetzke and Richards, 1999). Early vigor, in itself, could contribute to early establishment, weed suppression and overall improved assimilates balance. The delay in leaf growth of the GAI genotypes has also been reported to be associated with increased evaporation losses from the soil surface, which results in significant reduction of plant water-use efficiency (López-Castañeda and Richards, 1994). This pleiotropic effect of greater leaf area can benefit yield and has been proposed as a breeding target for semiarid regions (Richards et al., 2010; Rebetzke et al., 2014a).

The potential contribution to seedling establishment, of replacing the GAI dwarfing genes, has been extensively discussed (Rebetzke et al., 2012; Mohan et al., 2013). In the present study, however, we tested the possible effects on yield components. Grain yield is the final outcome of interrelated polygenically controlled developmental processes, including seedling establishment, tillering, floral initiation, anthesis, and grain filling. Deep sowing as well as genotype × environment interactions strongly affect these processes. In general, deep-sowing treatments had negative effects on the yield components associated with early wheat development (**Figure 4**). The GAR lines, with longer coleoptiles, emerged faster than the GAI ones from deep sowing and, consequently, their early yield components were significantly less affected. These differences in early development stages were evident in the differing final yield reductions noted in GAI and GAR: 37.3 and 12.1%, respectively (**Figure 4**; **Table 2**).

The advantage of GAR genotypes in their ability to emerge from deep sowing, as shown in the present study, was mostly expressed in the early growth period, whereas at later growth stages the effects of this delay in seedlings emergence on yield components were reduced (**Figure 4**). It seems that the faster leaf area development contributes to enhanced assimilation, which gives a significant advantage to the GAR genotypes, in developing more tillers and biomass than the GAI genotypes. Although this effect weakened in the course of time, it did have a significant impact on the final grain yield (**Figures 4** and **5**).

## Conclusion

Under Mediterranean-like conditions, the wheat-growing season is limited by the timing of autumn rains during germination and establishment, and by terminal drought during grain filling. Under such conditions, a delay of a few days in emergence from deep sowing have a long-term effect that results in a shortened growing season. Consequently, yield potential is reduced because grain filling occurs under suboptimal conditions. Our present results, as well as others, demonstrate the breeding potential of replacing the GAI dwarfing genes with GAR genes for improving wheat yields under drought conditions.

## Conflict of Interest Statement

The authors declare that the research was conducted in the absence of any commercial or financial relationships that could be construed as a potential conflict of interest.
