# Supplementary material for: Effect of GA-sensitivity on wheat early vigor and yield components under deep sowing
Source: Front Plant Sci. 2015 Jul 10;6:487. doi: 10.3389/fpls.2015.00487 (PMC4498040; doi:10.3389/fpls.2015.00487)
Supplement: Supplementary file 2 [file Presentation_1.PDF]

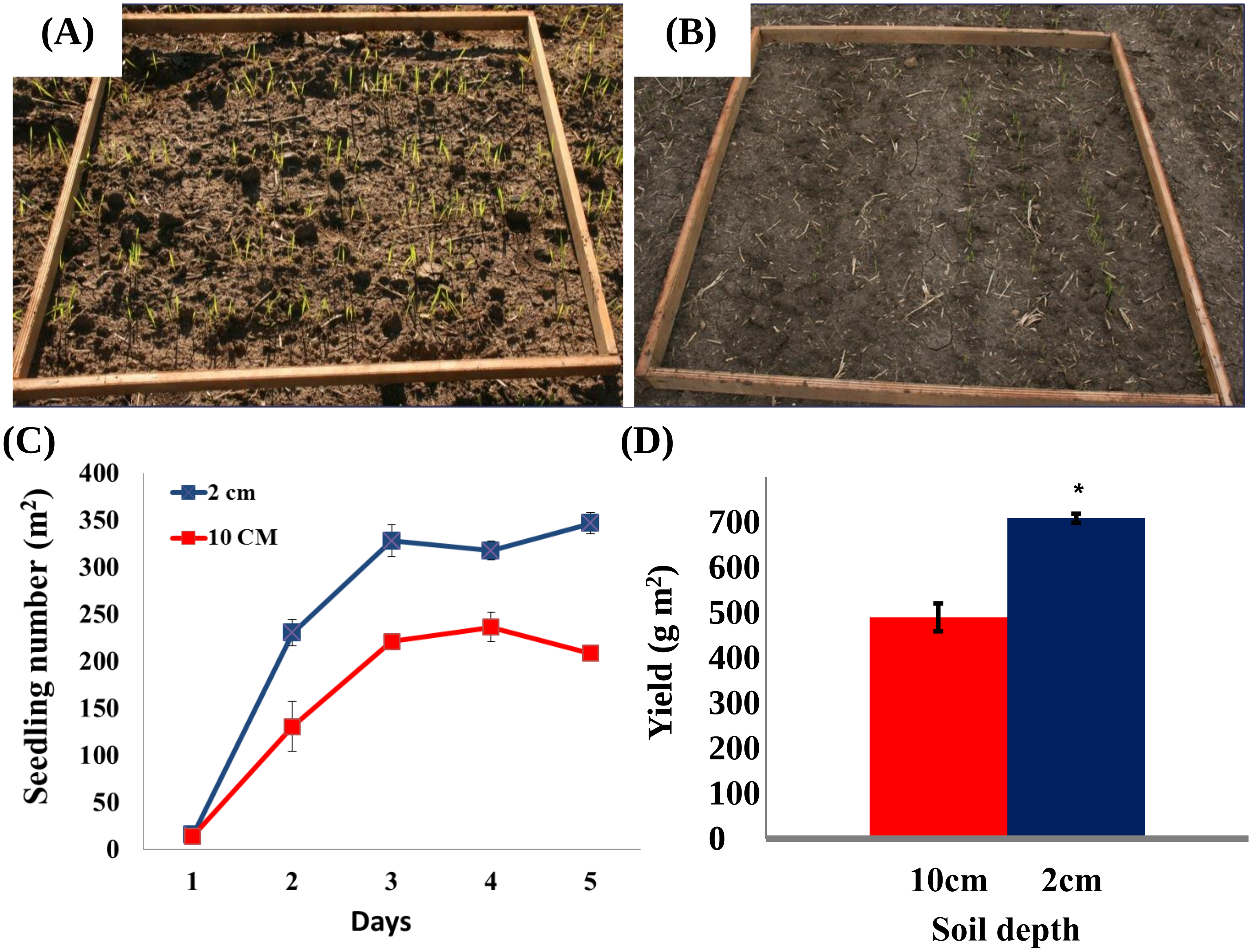

Supplementary Figure S1. Effect of sowing depth on seedling establishment in commercial field (cultivar Shaphir). Comparison of field stand (1 m^2) for (A) 2cm depth and (B) 10cm depth. (C) Number of seedling in 1m^2 plot and (D) Grain yield. * indicate significant differences between soil depths at $P \leq 0.05$.

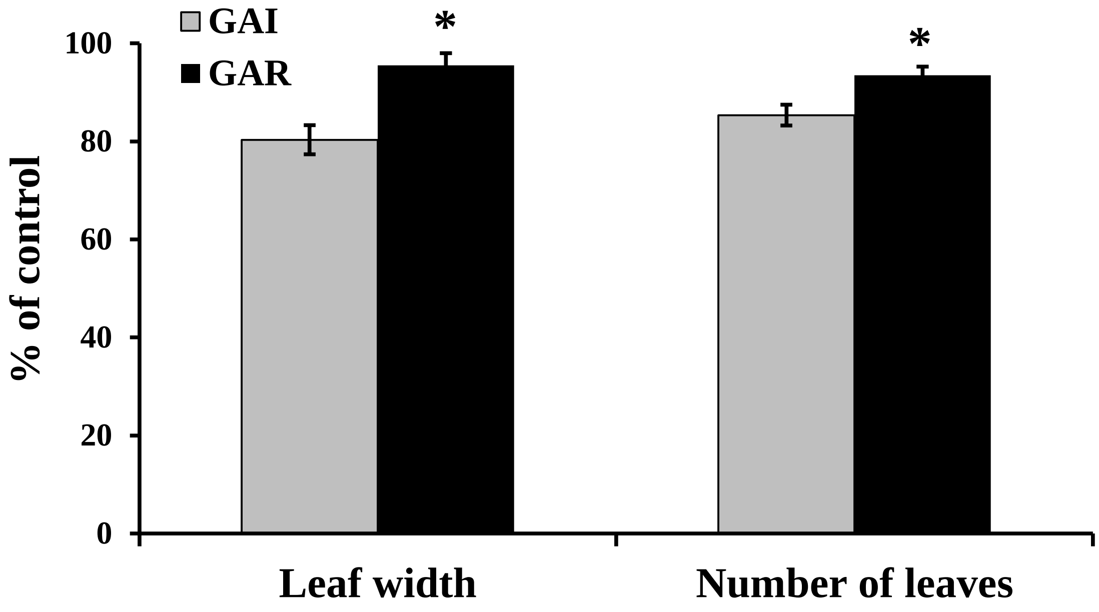

Supplementary Figure S2. Effect of sowing depth on seedling leaf width and the number of leaves. * indicate significant differences between the two soil depths at $P \leq 0.05$.

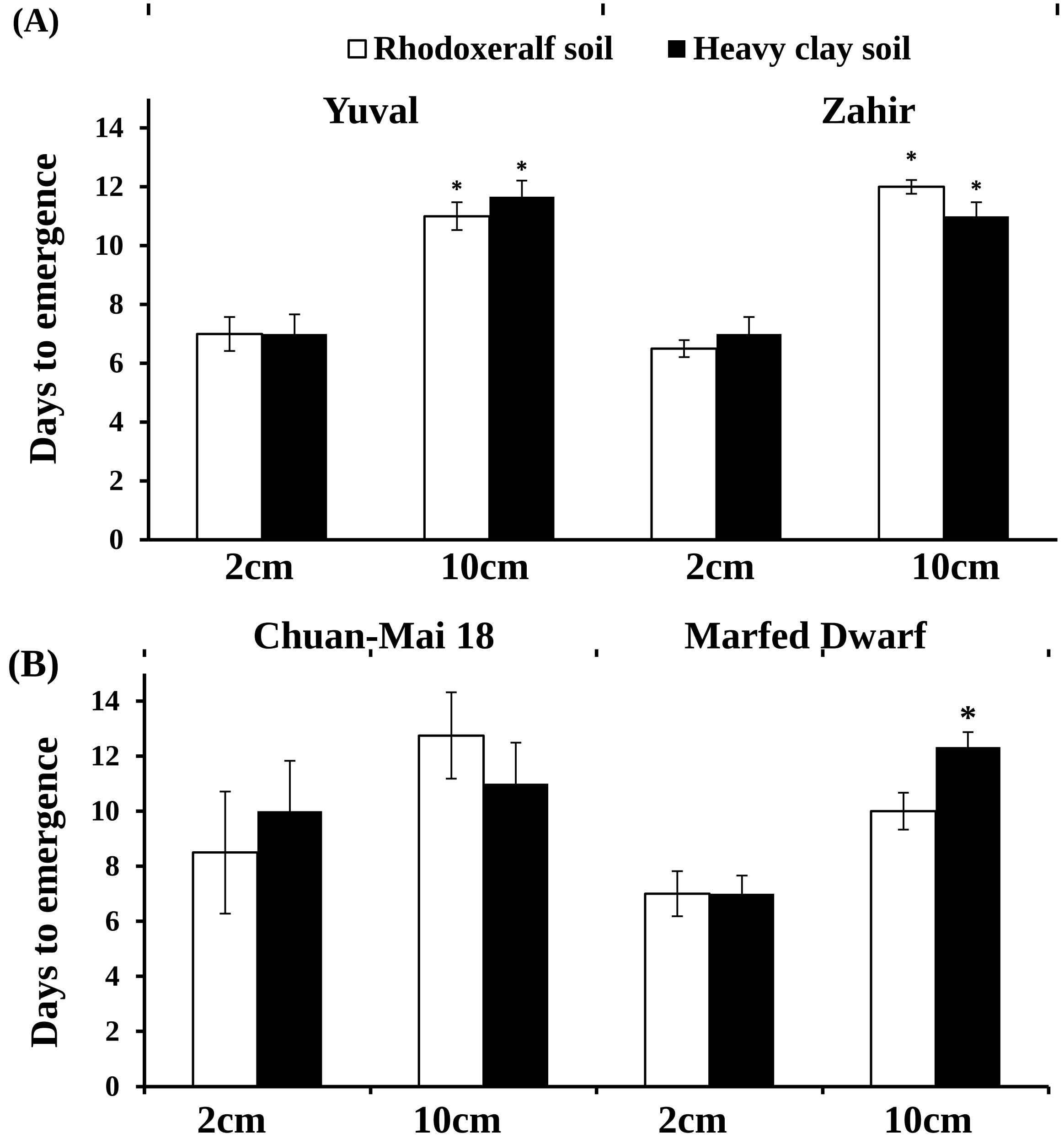

Supplementary Figure S3. Days to emergence of wheat genotypes under different soil types. (**A**) GAI lines- Israeli elite wheat cultivars (Yuval and Zhair respectively). (**B**) GAR lines- wheat lines (Marfed Dwarf and Chuan Mai-18, respectively). * indicate significant differences between soil depths at $P \leq 0.05$.
